# Effectiveness of malaria control interventions in Madagascar: a nationwide case–control survey

**DOI:** 10.1186/s12936-016-1132-x

**Published:** 2016-02-11

**Authors:** Thomas Kesteman, Milijaona Randrianarivelojosia, Vaomalala Raharimanga, Laurence Randrianasolo, Patrice Piola, Christophe Rogier

**Affiliations:** Malaria Research Unit, Institut Pasteur de Madagascar, BP 1274, 101 Avaradoha, Antananarivo, Madagascar; Unité de recherche sur les maladies infectieuses et tropicales émergentes (URMITE) - UMR 6236, 27 boulevard Jean Moulin, 13385 Marseille Cedex 05, France; Fondation Mérieux, 17 rue Bourgelat, 69002 Lyon, France; Epidemiology Unit, Institut Pasteur de Madagascar, BP 1274, 101 Avaradoha, Antananarivo, Madagascar; Institute for Biomedical Research of the French Armed Forces (IRBA), BP 73, 91223 Brétigny-Sur-Orge Cedex, France

**Keywords:** Malaria, Morbidity, Prevention and control, Case–control studies, Health surveys, Vector control, Insecticide-treated bed nets

## Abstract

**Background:**

Madagascar, as other malaria endemic countries, depends mainly on international funding for the implementation of malaria control interventions (MCI). As these funds no longer increase, policy makers need to know whether these MCI actually provide the expected protection. This study aimed at measuring the effectiveness of MCI deployed in all transmission patterns of Madagascar in 2012–2013 against the occurrence of clinical malaria cases.

**Methods:**

From September 2012 to August 2013, patients consulting for non-complicated malaria in 31 sentinel health centres (SHC) were asked to answer a short questionnaire about long-lasting insecticidal nets (LLIN) use, indoor residual spraying (IRS) in the household and intermittent preventive treatment of pregnant women (IPTp) intake. Controls were healthy all-ages individuals sampled from a concurrent cross-sectional survey conducted in areas surrounding the SHC. Cases and controls were retained in the database if they were resident of the same communes. The association between Plasmodium infection and exposure to MCI was calculated by multivariate multilevel models, and the protective effectiveness (PE) of an intervention was defined as 1 minus the odds ratio of this association.

**Results:**

Data about 841 cases (out of 6760 cases observed in SHC) and 8284 controls was collected. The regular use of LLIN provided a significant 51 % PE (95 % CI [16–71]) in multivariate analysis, excluding in one transmission pattern where PE was −11 % (95 % CI [−251 to 65]) in univariate analysis. The PE of IRS was 51 % (95 % CI [31–65]), and the PE of exposure to both regular use of LLIN and IRS was 72 % (95 % CI [28–89]) in multivariate analyses. Vector control interventions avoided yearly over 100,000 clinical cases of malaria in Madagascar. The maternal PE of IPTp was 73 %.

**Conclusions:**

In Madagascar, LLIN and IRS had good PE against clinical malaria. These results may apply to other countries with similar transmission profiles, but such case–control surveys could be recommended to identify local failures in the effectiveness of MCI.

**Electronic supplementary material:**

The online version of this article (doi:10.1186/s12936-016-1132-x) contains supplementary material, which is available to authorized users.

## Background

Malaria control has been intensified in the last decade, leading to an important reduction in its incidence [1]. In Madagascar, the incidence of clinical malaria in outpatient wards declined by 81 %, in inpatient wards by 69 % and mortality by 75 % between 2000 and 2010 [2]. This reduction in malaria burden could be attributed to the intensification and scale up of malaria control interventions (MCI), but it remains unknown what part of this decline was attributable to the MCIs. It is also not known which intervention(s) within the package is(are) most effective [3]. Moreover, other factors may have played a role that would balance the optimism arising from this success, e.g. increase of urbanization [4], climate change [5], and changes in socio-economic factors [6]. Roll-out of rapid diagnostic tests (RDT) may cause a false impression of decline in incidence because false positive cases—formerly diagnosed on the sole base of symptoms—are excluded from cases count [7]. Now that international funding for malaria control stopped increasing, it becomes of public health and strategic importance to measure the actual effectiveness of MCIs, in order to fund what is really working. In this context, the present study was aimed to evaluate whether interventions actually deployed performed well by measuring their individual effectiveness, their coverage and, by multiplication of these two values, their community effectiveness [8, 9]. Although the level of evidence generated by observational studies don’t reach the one generated by randomized controlled trials (RCTs) it would be unethical to conduct RCTs—and thus intentionally leave individuals unprotected—for policy guidance.

The study was named MEDALI—a French acronym standing for *Mission d’Etude des Déterminants de l’Accès aux Méthodes de Lutte antipaludique et de leur Impact*—and took place in Madagascar in 2012–2013. The design and methodology of the overall MEDALI project has been previously described [10]. The primary objective of the study presented here was the evaluation of the effectiveness against incidence of non-complicated clinical malaria of long-lasting insecticidal nets (LLIN) and indoor residual spraying (IRS) in the overall population. The secondary objective was the evaluation of the effectiveness against incidence of non-complicated clinical malaria of intermittent preventive treatment in pregnancy (IPTp) in a population of pregnant women or women having recently delivered.

## Methods

### Study design

**Fig. 1:**
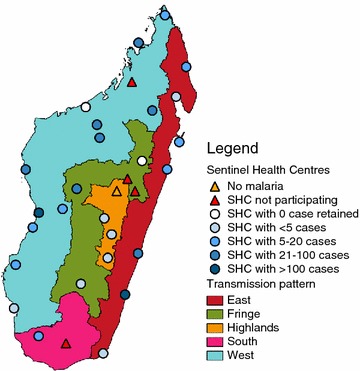
SHC and malaria transmission patterns in Madagascar

Districts of Madagascar are divided into five main operational zones (Fig. 1), which correspond to the transmission patterns of Madagascar [11]. The two coastal regions exhibit hyperendemic patterns with a transmission lasting all year in the East and more than 6 months per year in the West. In the central highlands, the transmission is unstable, and episodic or epidemic. In the fringe areas, i.e. at intermediate altitudes, the transmission pattern is seasonal, lasting from November to May (rainy season). In the South, the period of transmission is short and episodic. Fringe, central highlands and South are prone to outbreaks.

The selection of study sites was based on a network of sentinel health centres (SHC) for surveillance of fever-associated diseases that has been established in order to cover all the ecosystems of Madagascar [12]. Each location where at least one SHC existed in 2012 was included in the study, thus defining 31 study sites. All malaria transmission patterns were represented: 13 sites were located in the Western transmission pattern, seven in the East, five in the Fringe, four in the Central Highlands and two in the South (Fig. 1). These patterns encompass respectively 21.0, 27.5, 13.7, 31.9 and 5.9 % of Malagasy population. The design consisted in recruiting non-complicated clinical malaria cases in health facilities belonging to the SHC network, and controls in the population living in their catching same areas.

### Inclusion of cases

All 31 SHCs were proposed to participate in the study protocol. In the participating SHCs, patients presenting with clinical malaria cases or their tutors were asked to answer a short one-page questionnaire about socio-demographic data and exposure to MCIs: LLIN, IRS and IPTp. Inclusion criteria were (1) fever, i.e., axillary temperature ≥37.5 °C [13] or self-reported symptoms of fever; (2) RDT or microscopy positive for any *Plasmodium* species; (3) age ≥6 months; and (4) informed consent of the patient or his/her tutor. CareStart^®^ Malaria RDT (Access Bio Inc., Monmouth Junction (NJ), USA) was used, which is the RDT commonly used in the public health system in Madagascar. Cases were retained in the database if they came from the same commune as controls. Data were collected from September 2012 to August 2013.

### Inclusion of controls

Controls were selected from a cross-sectional survey (CSS), which took place in the same areas in the context of the very same MEDALI project, between September 2012 and January 2013. The methodology of this CSS has been previously described [10]. Briefly, it was a cross-sectional household survey in which a two-stage cluster sampling technique was used to randomly select two *fokontanys* (smallest administrative subdivision in Madagascar) near each SHC. In each *fokontany*, the investigators followed a random path to include 50 households, i.e. approximately 225 individuals per site. The sample size of controls was calculated for a concomitant cross-sectional survey, leaving controls in excess [10, 14, 15]. Heads of households and all members of the household eligible for the survey were interviewed about socio-demographic features and exposure to MCIs, their axillary temperature was measured, and a RDT (CareStart^®^ Malaria) was performed. Inclusion criteria were: (1) age ≥6 months, (2) having signed individual informed consent including agreement for blood sampling, and (3) being able to take a per os treatment in case of positive RDT. Parents or tutors signed and answered the questionnaire for minors and impaired individuals. Controls were retained in the database if they came from the same commune as cases, if they were permanent residents of the household, and if they had no malaria at the time of survey (i.e. fever or history of fever, and RDT or microscopy positive for any *Plasmodium* species), or in the last 3 months (i.e. diagnosis of malaria, or history of fever treated with anti-malarial drugs, or history of fever with unknown management).

### Sample size calculation

The primary objective for sample size calculation was to detect the association between occurrence of clinical malaria due to any *Plasmodium* species, and exposure to MCI. It was assumed that at least three controls would be found for each case. A sample size of 800 cases and 2400 controls has a power of 87, 70, and 49 % for detecting OR of 0.7, 0.75 and 0.8, respectively, considering the following parameters: coverage of intervention of 50 % in controls, cluster effect of 2, and alpha risk of 5 % [16].

### Data management and statistical analyses

Bed net use was defined as “use every night during last 3 months” because it is more stringent than the “last night” definition [10, 14]. The association between exposure to MCIs and being a case was estimated by generalized estimating equations models (GEE) taking into account an exchangeable within-site correlation structure using *gee* function on R software [17]. GEE models allow for a robust estimation of ORs and their confidence intervals while controlling for clustering [9]. Controls were neither matched with cases nor limited to three controls per case, but adjustment variables (age, sex, and transmission pattern) were forced in all models. All multivariate model fits were evaluated using binned residual plots [18, 19]. Whether malaria transmission pattern, age less than 5 years, or season of detection of cases influenced the effectiveness of MCI was tested by introducing interaction terms in the models. Whenever season modified the effectiveness measured, the analysis was rerun on the cases that occurred in the same quarter as the collection of data on controls. The protective effectiveness (PE) of an intervention was defined as 1 minus the odds ratio of the exposure to this intervention as suggested previously [8].

### Estimation of the number of clinical cases of malaria avoided

In order to evaluate the number of clinical cases of malaria prevented by each MCI with significant PE, the PE value was first multiplied by the coverage of the MCI in the general population, thus giving the proportion of cases avoided or “community effectiveness” (CE), as described previously [8]. The estimated number of cases avoided was defined as the annual number of clinical cases multiplied by CE/(1 − CE). Coverage values were extracted from the concomitant CSS mentioned previously [10], and number of malaria cases in 2011 by districts was provided by the National Malaria Control Programme.

### Ethical considerations

All surveys followed ethical principles according to the Helsinki Declaration. Informed consent was obtained from the individuals, or the parents/tutors of the children before inclusion. The protocol was approved by the National Ethic Committee of the Ministry of Public Health of Madagascar (approval #CNE 57/MSANP/CE of July 24th, 2012).

## Results

**Fig. 2:**
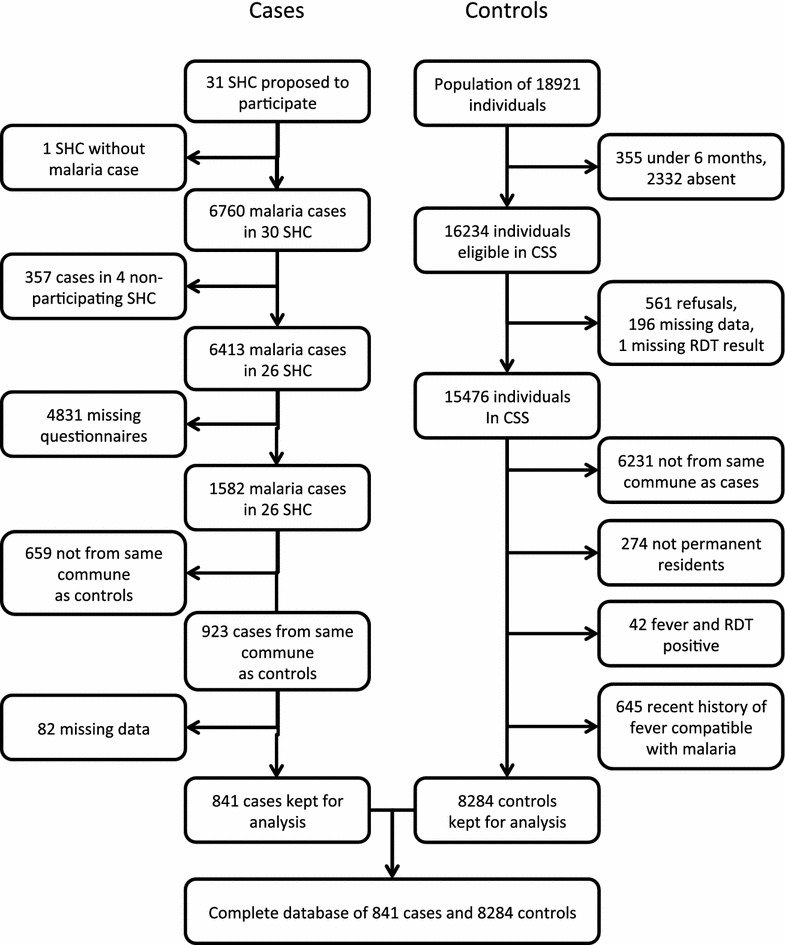
Flow diagram

From September 2012 to August 2013, 6760 clinical malaria cases were observed in 30 SHCs. Four SHC did not participate and one had no malaria cases during the period (Figs. 1, 2). Among the remaining 6413 cases, 1582 questionnaires (24.7 %) were filled. The proportion of refusals among missing questionnaires is unknown. Individuals coming from the same communes as individuals included in the CSS accounted for 58.3 % of the questionnaires sent. Among those, 841 questionnaires (91.1 %) were correctly filled, from 24 SHC (Fig. 2). Overall, 34 clusters (communes) were identified in all transmission patterns: 61.4 % of cases occurred in the West, 30.0 % in the East, 6.9 % in the Fringe, 1.0 % in the South, and 0.8 % in the Highlands. The mean number of clinical malaria cases per cluster was 24.7 individuals (range 1–179). These episodes occurred between the end of September 2012 and mi-June 2013: 39.3 % in spring, i.e. before December 31st, 2012; 47.8 % in summer, i.e. between January 1st and March 31st, 2013; and 12.9 % in fall, i.e. after April 1st, 2013.

In a population of 18,921 individuals, 15,476 were included in the CSS [10, 14]. Among those, 6231 (40.3 %) were not comparable to cases since they were located in other communes, and 274 (1.8 %) were not permanent resident of the households where they had been identified. Of the remaining 8971 individuals, 42 (0.5 %) had an ongoing malaria episode and 645 (7.2 %) described a history of fever within the last 3 months that was compatible with an episode of malaria (Fig. 2). The study ended up with a database of 841 cases and 8284 controls, dispatched in 34 clusters (communes). The majority of the sample was from the Western transmission pattern (61.4 % of cases, 52.2 % of controls), then came the East (30.0 % of cases and 26.5 % of controls), and Fringe (6.9 % of cases and 8.1 % of controls); the Highlands and the Southern transmission patterns encompassed seven and eight cases only (representing 0.8 and 1.0 % of cases, respectively) and their controls represented 8.1 and 5.1 % of controls, respectively.

### Bed nets

In the areas where LLIN are distributed, i.e. Eastern, Western, Southern, and Fringe transmission patterns, the sample encompassed 31 clusters including 834 cases and 7617 controls. The use of LLIN every night was higher in controls (53.2 %) than in cases (39.3 %), and provided a significant 53 % PE (95 % CI [20–73]) in bivariate and 50 % (95 % CI [16–70]) in multivariate analyses (Additional file 1). No significant interaction term was found between the season or the age under than 5 years old, and LLIN use. LLIN use among cases decreased from 43.3 % in spring, to 37.4 % in summer, and to 34.4 % in fall, but this difference was not significant (Fisher’s exact test, p > 0.1). A significant interaction term between the southern transmission pattern and LLIN use was identified (p < 0.001), and separate analyses were conducted for the South and the rest of target zones. In the South, the association between LLIN use and clinical malaria was non- significant (OR 1.11, 95 % CI [0.35–3.51]) in bivariate analysis. The small number of cases (n = 8) in the South precluded the possibility to conduct a multivariate analysis. In all other areas targeted by LLIN distribution campaigns, the use of LLIN every night provided a significant 51 % PE (95 % CI [16–71], Table 1), and the use of non-impregnated bed net (NIBN) a significant 37 % PE in multivariate analysis (95 % CI [6–58], Table 1).

**Table 1:** Bi- and multivariate analyses of risk factors for developing a clinical malaria episode, including bed net use, in multivariate analyses, in zones targeted for LLIN distribution excluding the Southern transmission pattern (826 cases, 7192 controls, 29 communes)

Variable	Category	N cases (%)	N controls (%)	Bivariate		Multivariate	
				Crude OR [95 %CI]	p	Adjusted OR [95 %CI]	p
Every night bed net use	LLIN	326 (39.5)	3959 (55.0)	0.47 [0.27–0.80]	0.005	0.49 [0.29–0.84]	0.009
	NIBN	84 (10.2)	857 (11.9)	0.62 [0.40–0.95]	0.029	0.63 [0.42–0.94]	0.023
	None	416 (50.4)	2376 (33.0)	1.00		1.00	
Age group	0–1 year	56 (6.8)	302 (4.2)	3.42 [1.53–7.64]	0.003	3.29 [1.45–7.48]	0.004
	2–4 years	113 (13.7)	771 (10.7)	2.93 [1.66–5.17]	<0.001	2.72 [1.51–4.90]	<0.001
	5–9 years	175 (21.2)	1228 (17.1)	2.70 [1.79–4.06]	<0.001	2.44 [1.63–3.65]	<0.001
	10–15 years	169 (20.5)	1051 (14.6)	2.97 [2.12–4.14]	<0.001	2.57 [1.85–3.58]	<0.001
	15–19 years	122 (14.8)	762 (10.6)	3.01 [1.93–4.69]	<0.001	2.49 [1.66–3.75]	<0.001
	20–39 years	137 (16.6)	1784 (24.8)	1.67 [0.98–2.87]	0.061	1.57 [0.93–2.65]	0.088
	≥40 years	54 (6.5)	1294 (18.0)	1.00		1.00	
Sex	Male	447 (54.1)	3114 (43.3)	1.00		1.00	
	Female	379 (45.9)	4078 (56.7)	0.64 [0.53–0.78]	<0.001	0.74 [0.62–0.87]	<0.001
Transmission pattern	East	252 (30.5)	2194 (30.5)	1.10 [0.16–7.55]	0.923	1.90 [0.24–15.13]	0.542
	Fringe	58 (7.0)	670 (9.3)	1.00		1.00	
	West	516 (62.5)	4328 (60.2)	1.41 [0.27–7.44]	0.686	1.46 [0.22–9.60]	0.696

### IRS

IRS campaigns took place in the Fringe, in most of Highlands, and in certain parts of Western and Southern transmission patterns. In these areas, living in a household that had been sprayed within the last 12 months provided a significant 54 % PE in bivariate and multivariate analyses (95 % CI [30–70] and [36–68], respectively). Models were run on a database of 11 clusters, including 199 cases and 2680 controls. No significant interaction term between the age under than 5 years old, or transmission pattern, and exposure to IRS was found. IRS coverage in the previous year among cases significantly decreased from 72.5 % in the spring, to 43.5 % in the summer, and to 4.8 % in the fall (Fisher’s exact test, p < 0.001). Interaction terms between season and IRS were also significant (p < 0.001), and the final models were thus restricted to cases having occurred in the spring of 2012. In this analysis, 179 cases and 1125 controls belonging to five clusters in the Fringe, Western and Southern transmission patterns were analyzed. The PE of IRS was 50 % in bivariate analysis (95 % CI [24–67]) and 51 % in multivariate analysis (95 % CI [31–65], Table 2).

**Table 2:** Bi- and multivariate analyses of risk factors for developing a clinical malaria episode, including IRS the previous year in the household (179 cases, 1125 controls, 5 communes)

Variable	Category	N cases (%)	N controls (%)	Bivariate		Multivariate	
				Crude OR [95 %CI]	p	Adjusted OR [95 %CI]	p
IRS the previous year	No	92 (51.4)	778 (69.2)	0.50 [0.33–0.76]	0.001	0.49 [0.35–0.69]	<0.001
	Yes	87 (48.6)	347 (30.8)	1.00		1.00	
Age group	0–1 year	19 (10.6)	37 (3.3)	26.08 [12.00–56.71]	<0.001	20.76 [9.44–45.66]	<0.001
	2–4 years	34 (19.0)	130 (11.6)	14.24 [6.12–33.16]	<0.001	12.85 [5.77–28.63]	<0.001
	5–9 years	38 (21.2)	198 (17.6)	11.17 [5.39–23.16]	<0.001	9.69 [5.07–18.52]	<0.001
	10–15 years	26 (14.5)	180 (16.0)	7.33 [2.08–25.90]	0.002	6.44 [2.03–20.41]	0.002
	15–19 years	26 (14.5)	125 (11.1)	11.29 [3.57–35.67]	<0.001	10.03 [3.93–25.58]	<0.001
	20–39 years	33 (18.4)	247 (22.0)	7.34 [2.77–19.43]	<0.001	6.45 [2.81–14.81]	<0.001
	≥40 years	3 (1.7)	208 (18.5)	1.00		1.00	
Sex	Male	98 (54.7)	476 (42.3)	1.00		1.00	
	Female	81 (45.3)	649 (57.7)	0.59 [0.52–0.66]	<0.001	0.65 [0.55–0.78]	<0.001
Transmission pattern	Fringe	55 (30.7)	219 (19.5)	1.00		1.00	
	West	118 (65.9)	697 (62.0)	0.61 [0.25–1.47]	0.269	1.50 [0.71–3.14]	0.288
	South	6 (3.4)	209 (18.6)	0.11 [0.11–0.11]	<0.001	0.13 [0.12–0.14]	<0.001

### Concurrent exposure to IRS and LLIN

Given the above reported interactions, the analysis was restricted to cases occurring in the spring of 2012, and excluded the Southern transmission pattern, resulting in a sub-sample comprising four clusters, 173 cases and 916 controls in the Fringe and Western transmission patterns. In these clusters where IRS campaigns and LLIN distributions occurred, being exposed to both MCI provided a significant 72 % PE in bivariate and multivariate analyses (95 % CI [22–90] and [28–89], respectively, Table 3). There was some association between exposure to both intervention and exposure to a single intervention, but it did not reach the significance threshold (OR against LLIN alone 0.75, 95 % CI [0.54–1.04], p = 0.082, and OR against IRS alone 0.63, [0.38–1.06], p = 0.082).

**Table 3:** Bi- and multivariate analyses of risk factors for developing a clinical malaria episode, including bed net use and IRS (134 cases, 749 controls, 4 communes)

Variable	Category	N cases (%)	N controls (%)	Bivariate		Multivariate	
				Crude OR [95 %CI]	p	Adjusted OR [95 %CI]	p
Every night bed net use and/or IRS the previous year	LLIN use and IRS	28 (16.2)	335 (36.6)	0.28 [0.10–0.78]	0.015	0.28 [0.11–0.72]	0.008
	No LLIN use and IRS	60 (34.7)	317 (34.6)	0.43 [0.25–0.74]	0.003	0.44 [0.26–0.74]	0.002
	LLIN use and no IRS	12 (6.9)	97 (10.6)	0.34 [0.10–1.11]	0.073	0.37 [0.11–1.25]	0.110
	NIBN use and no IRS	6 (3.5)	18 (2.0)	1.00 [0.78–1.30]	0.972	0.90 [0.75–1.09]	0.287
	No bed net use and no IRS	67 (38.7)	149 (16.3)	1.00		1.00	
Age group	0–1 year	19 (11.0)	31 (3.4)	5.69 [3.35–9.69]	<0.001	4.78 [2.56–8.92]	<0.001
	2–4 years	33 (19.1)	96 (10.5)	3.12 [2.33–4.18]	<0.001	3.02 [1.96–4.65]	<0.001
	5–9 years	38 (22.0)	152 (16.6)	2.49 [1.95–3.19]	<0.001	2.38 [1.81–3.12]	<0.001
	10–15 years	23 (13.3)	148 (16.2)	1.36 [0.83–2.23]	0.218	1.27 [0.70–2.31]	0.440
	15–19 years	24 (13.9)	111 (12.1)	2.17 [1.52–3.09]	<0.001	1.97 [1.48–2.62]	<0.001
	≥20 years	36 (20.8)	378 (41.3)	1.00		1.00	
Sex	Male	94 (54.3)	386 (42.1)	1.00		1.00	
	Female	79 (45.7)	530 (57.9)	0.59 [0.54–0.66]	<0.001	0.68 [0.56–0.83]	<0.001
Transmission pattern	Fringe	55 (31.8)	219 (23.9)	1.00		1.00	
	West	118 (68.2)	697 (76.1)	0.61 [0.25–1.47]	0.268	1.14 [0.94–1.38]	0.196

### IPTp

In Madagascar, IPTp is proposed in all transmission patterns except in the Highlands. In these areas, having taken at least one dose of IPTp during pregnancy provided a 78 % PE in bivariate analysis and a 73 % PE in multivariate analysis, but these results were not statistically significant (Table 4).

**Table 4:** Bi- and multivariate analyses of risk factors for developing a clinical malaria episode, including IPTp

Variable	Category	N cases (%)	N controls (%)	Bivariate		Multivariate	
				Crude OR [95 %CI]	p	Adjusted OR [95 %CI]	p
≥1 dose IPTp	Yes	3 (25.0)	11 (57.9)	0.22 [0.04–1.29]	0.093	0.27 [0.04–1.89]	0.187
	No	9 (75.0)	8 (42.1)	1.00		1.00	
Age group	15–24 years	11 (91.7)	8 (42.1)	14.24 [4.02–50.38]	<0.001	12.35 [3.90–39.11]	<0.001
	25–49 years	1 (8.3)	11 (57.9)	1.00		1.00	

Transmission patterns could not be included because it caused the model not to converge (12 cases, 110 controls, 5 communes)

### Estimation in the number of clinical malaria cases avoided by LLIN and IRS at the country level

Considering number of malaria cases provided by National Malaria Control Programme for 2011 and coverage from the CSS mentioned previously [10], these interventions could have saved over 100,000 clinical malaria cases in one year (Table 5), 88 % by LLIN and 12 % by IRS.

**Table 5:** Estimation of the annual number of clinical malaria cases prevented by vector control interventions in Madagascar

Transmission pattern	N cases in 2011	LLIN				IRS				Total N cases prevented
		PE (%)	Cov. (%)	CE (%)	N cases prevented	PE (%)	Cov. (%)	CE (%)	N cases prevented	
East	157,858	51	61.1	31	71,457	NA	3.8	NA	NA	71,457
Fringe	8347	51	35.4	18	1839	51	58.6	30	3558	5397
West	40,068	51	55.7	28	15,898	51	18.3	9	4124	20,023
South	8481	−30	41.9	NA	NA	51	67.8	35	4483	4483
Highlands	6297	NA	7.7	NA	NA	51	43.1	22	1774	1774
Total	221,051				89,194				12,165	101,359

*PE* protective effectiveness, *CE* community effectiveness, *NA* not applicable

## Discussion

During the last decade, Madagascar has started and scaled up various interventions and policy changes regarding malaria control, resulting in dramatic decline in malaria burden, but the impact of each MCI cannot be disentangled by ecological studies [3]. This case–control study provided reliable information for guidance in policy making. In this study, vector control interventions demonstrated important and significant PE: LLIN provided a 51 % PE (95 % CI 16–71 %) and IRS a 51 % PE (95 % CI 31–65) when controlling for major confounding variables.

Deployment of LLIN and IRS may have prevented 100,000 cases annually. Note that IRS in Madagascar is mainly deployed in low-transmission settings and LLIN in almost all the country, which explains why the number of prevented cases differs so much between the two MCI while PE are similar. Even though it may be regarded as simplistic to add cases avoided by LLIN to cases avoided by IRS, these results give a rough overview of the number of prevented cases, and they could feed more sophisticated cost-effectiveness or transmission models.

The present results are close to results from a simultaneous CSS survey aimed at estimating the effectiveness of MCI against *Plasmodium* infection where LLIN was found to have a 41 % PE, IRS had a 44 % PE at household level in certain places, the concurrent exposure to both LLIN and IRS had a 86 % PE, and IPTp a 65 % PE [14, 15]. This suggests that measuring the effectiveness of MCI for one outcome, e.g. malaria infection is a reasonable proxy for the other, e.g. clinical malaria. As observed in the study mentioned above, the southern part of Madagascar appears to be different from the other zones, but the present study lacked statistical power to evaluate the effectiveness of LLIN in this transmission pattern since the sample size of cases was eight only.

The results from the present study are also in line with results from efficacy studies of LLIN and IRS, which suggests that the efficacy of MCI is preserved in Madagascar. In meta-analyses, insecticides-treated nets (ITN) were shown to provide a 50 % PE against malaria incidence as compared to no nets in areas of stable malaria, and a 62 % PE in areas of unstable malaria [20], and the PE of IRS ranged between 31 and 88 % in areas of unstable malaria [21]. Nevertheless, in contrast with these studies, the present results show no relationship between transmission patterns and MCI effectiveness. This might be explained by an insufficient statistical power in the present study to detect such a difference, since few cases have been identified in low-transmission areas. In the present study, the age under 5 years was not associated with a better effectiveness of MCI, while this is theoretically expected [22] and observed in some efficacy studies [23].

This study design was suggested more than a decade ago in order to monitor the effectiveness of MCI through time and space [8, 9], but relatively few surveys have been conducted. The scarceness of CCS is surprising given the relative simplicity of the survey design and importance of the information it provides in terms of policy guidance. If vector control interventions are consider, for example, only 13 CCS have tried to measure the PE of ITN [9, 24–34], and four the PE of IRS—including two of the previous [24, 25, 35, 36]. Only six of these 15 surveys collected data in a geographical region of the size of a district or above, and their sample sizes were smaller (mean number of cases 219, range 35–534), which makes the present study the biggest CCS ever aimed at evaluating the effectiveness of MCI, with a unique countrywide design.

Nine of the CCS mentioned above found a significant PE of ITN. As compared with these studies, the use of bed net was defined more restrictively, i.e. the use every night in the last 3 months instead of the use last night. This definition was selected in order to provide a more accurate estimation of the effectiveness in the perspective of being used for mathematical modelling or for calculation of the cost-effectiveness ratio. Indeed, the “last night” definition is more sensitive to social desirability bias and clerical errors, and tends to overestimate the coverage [10].

This study presents several limitations. One of the main issues is that a minimal questionnaire was used in order to facilitate the practitioners’ task, preventing us from collecting useful data such as socioeconomic status or education level for adjustment of multivariate models. Nevertheless, in a concurrent study aimed at evaluating the effectiveness of the very same MCI against infection through a CSS design, the difference between crude and adjusted OR were trivial.

The results of the study might have underestimated the actual effectiveness of LLIN and IRS for four reasons. Firstly, the study design does not allow for calculation of herd protection offered by vector control interventions while some authors claim that the protection offered by these interventions is more visible at the community than at the individual or household level, especially IRS [37]. Secondly, the survey did not cover the period during which IRS is expected to have an effect on vectors, i.e. a few months after spraying [38]. The previous IRS campaigns took place in November–December 2011, and one happened at the end of the period of data collection, in March–April 2013. The latter campaign was supposed to take place in November–December 2012, but was delayed. The low coverage (i.e. household sprayed last 12 months) of cases in summer might thus reflect a long interval since the previous IRS campaign, and the PE derived from these cases is probably not reliable. The most comparable cases with controls are definitely those who have been identified in the spring of 2012, i.e. at the same moment as the CSS. When the analysis was restrained to these individuals, the PE of IRS was demonstrated. As other authors have shown, the duration of the bio-efficacy of IRS was not expected to last more than 6–8 months [38], but its impact on malaria incidence may remain after the disappearance of the insecticide effect [14, 39, 40]. Third, LLIN use in controls was estimated in a season where bed net use was low [10, 41]. Nevertheless, non-significant interaction terms indicate that season did not influence the measure of the effectiveness of LLIN. Fourth, a recall bias might have occurred, as for all case–control studies. If cases better recalled exposure to MCIs, or if memory failures occurred in both cases and controls, then hypothesis tests would be biased towards the null [42].

Another limitation is the selection of cases and controls. In a theoretical database of over 6000 malaria cases, only 841 were retained, mainly because practitioners who agreed to participate in the study did not systematically fill in questionnaires. Nevertheless, since the choice of submitting the questionnaire to the patient or not was likely not driven by the exposure to MCI or confounding factors related to the place, one can reasonably suppose that no systematic bias occurred. Regarding controls, they are not strictly comparable to cases since they have not been selected in the operational catchment population of SHC, i.e. the individuals who get to the SHC when they are affected by febrile illnesses, but in the geographical catchment population, i.e. living in the same commune as cases. Typically, coverage of the three MCI investigated here were higher in less populated areas [10] who have a likely lower access to health facilities; this might have resulted in an overestimation of the effectiveness of MCI. Recruiting controls among non-malaria patients or persons accompanying patients seen in the same health structures as patients would avoid this bias.

Finally, the present study has serious limitations in its statistical power regarding IPTp since the sample sizes are small. Therefore, the non-significant OR of IPTp shall not be considered as reflecting a poor effectiveness but rather as the reflection of an insufficient statistical power. Moreover the short questionnaire did not allow for the collection of data about parity, which would have been preferable to adjust for. Similarly, the non-significant additional effect of both LLIN and IRS over one single intervention might be attributed to a lack of statistical power since the analysis has been conducted on a small subset of the whole database.

Despite these limitations, the strengths of this study—its large sample size, its countrywide scale, and its population-wide representativeness—suggest that the results are valuable for policy guidance in Madagascar. They allow, for the first time, an estimation of the number of clinical cases that have avoided by preventive measures. That opens the way for cost-effectiveness analysis that could help the health authorities in the choice of funding the MCI.

## Conclusions

The results of this study showed that, in Madagascar in 2012, both LLIN and IRS provided good protective effectiveness against clinical malaria. The study failed to prove that combining both MCI simultaneously might provide additional effect. However, combining these two vector control interventions might be indicated if the coverage or the effectiveness of one of them is problematic. The present study also could prompt stakeholders to fund operational research in other endemic countries, in order to verify the local performance of MCI by case–control approaches.

## Additional file

10.1186/s12936-016-1132-x Effectiveness of LLIN in all areas with LLIN distribution. Bi- and multivariate analyses of risk factors for developing a clinical malaria episode,including bed net use.

## Abbreviations

CCS: case–control study
CE: community effectiveness
CSS: cross-sectional survey
GEE: generalized estimating equations
IPTp: intermittent preventive treatment in pregnancy
ITN: insecticide-treated nets
IRS: indoor residual spraying
LLIN: long-lasting insecticidal nets
MCI: malaria control interventions
MEDALI: Mission d’Etude des Déterminants de l’Accès aux Méthodes de Lutte antipaludique et de leur Impact
NIBN: non-impregnated bed net
OR: odds ratio
PE: protective effectiveness
RCT: randomized controlled trial
RDT: rapid diagnostic tests
SHC: sentinel health centres
