# Supplementary material for: Effectiveness of malaria control interventions in Madagascar: a nationwide case–control survey
Source: Malar J. 2016 Feb 11;15:83. doi: 10.1186/s12936-016-1132-x (PMC4751752; doi:10.1186/s12936-016-1132-x)
Supplement: Supplementary file 1 — 10.1186/s12936-016-1132-x Effectiveness of LLIN in all areas with LLIN distribution. Bi- and multivariate analyses of risk factors for developing a clinical malaria episode,including bed net use. [file 12936_2016_1132_MOESM1_ESM.pdf]

Effectiveness of LLIN in all areas with LLIN distribution

Variable	Category	N cases (%)	N controls (%)	Bivariate		Multivariate	
				Crude OR [95% CI]	p	Adj. OR [95% CI]	p
**Every night bed net use**	LLIN	328 (39.3)	4052 (53.2)	0.47 [0.27 - 0.80]	0.006	0.50 [0.30 - 0.84]	0.009
	NIBN	84 (10.1)	874 (11.5)	0.62 [0.40 - 0.96]	0.032	0.63 [0.43 - 0.94]	0.023
	None	422 (50.6)	2691 (35.3)	1.00		1.00	
**Age group**	0-1 year	56 (6.7)	334 (4.4)	3.42 [1.53 - 7.62]	0.003	3.26 [1.45 - 7.36]	0.004
	2-4 years	114 (13.7)	841 (11.0)	2.96 [1.66 - 5.25]	<0.001	2.72 [1.51 - 4.88]	<0.001
	5-9 years	176 (21.1)	1323 (17.4)	2.72 [1.79 - 4.16]	<0.001	2.45 [1.64 - 3.65]	<0.001
	10-15 years	172 (20.6)	1114 (14.6)	3.05 [2.16 - 4.31]	<0.001	2.62 [1.88 - 3.65]	<0.001
	15-19 years	124 (14.9)	787 (10.3)	3.11 [1.96 - 4.94]	<0.001	2.56 [1.69 - 3.86]	<0.001
	20-39 years	138 (16.5)	1872 (24.6)	1.70 [0.98 - 2.95]	0.061	1.59 [0.94 - 2.67]	0.083
	≥40 years	54 (6.5)	1346 (17.7)	1.00		1.00	
**Sex**	Male	453 (54.3)	3283 (43.1)	1.00		1.00	
	Female	381 (45.7)	4334 (56.9)	0.64 [0.53 - 0.77]	<0.001	0.73 [0.61 - 0.86]	<0.001
**Transmission patterns**	East	252 (30.2)	2194 (28.8)	1.10 [0.16 - 7.55]	0.923	1.90 [0.24 - 15.01]	0.541
	Fringe	58 (7.0)	670 (8.8)	1.00		1.00	
	West	516 (61.9)	4328 (56.8)	1.41 [0.27 - 7.44]	0.686	1.49 [0.23 - 9.63]	0.678
	South	8 (1.0)	425 (5.6)	0.25 [0.04 - 1.37]	0.109	0.26 [0.02 - 3.97]	0.335

Bi- and multivariate analyses of risk factors for developing a clinical malaria episode, including bed net use.
